# Characterization of sialylation-related long noncoding RNAs to develop a novel signature for predicting prognosis, immune landscape, and chemotherapy response in colorectal cancer

**DOI:** 10.3389/fimmu.2022.994874

**Published:** 2022-10-18

**Authors:** Mingxuan Zhou, Silin Lv, Yufang Hou, Rixin Zhang, Weiqi Wang, Zheng Yan, Tiegang Li, Wenqiang Gan, Zifan Zeng, Fang Zhang, Min Yang

**Affiliations:** State Key Laboratory of Bioactive Substances and Function of Natural Medicine, Institute of Materia Medica, Chinese Academy of Medical Sciences and Peking Union Medical College, Beijing, China

**Keywords:** sialylation, long noncoding RNA, colorectal cancer, prognostic signature, immune microenvironment

## Abstract

Aberrant sialylation plays a key biological role in tumorigenesis and metastasis, including tumor cell survival and invasion, immune evasion, angiogenesis, and resistance to therapy. It has been proposed as a possible cancer biomarker and a potential therapeutic target of tumors. Nevertheless, the prognostic significance and biological features of sialylation-related long noncoding RNAs (lncRNAs) in colorectal cancer (CRC) remain unclear. This study aimed to develop a novel sialylation-related lncRNA signature to accurately evaluate the prognosis of patients with CRC and explore the potential molecular mechanisms of the sialylation-related lncRNAs. Here, we identified sialylation-related lncRNAs using the Pearson correlation analysis on The Cancer Genome Atlas (TCGA) dataset. Univariate and stepwise multivariable Cox analysis were used to establish a signature based on seven sialylation-related lncRNAs in the TCGA dataset, and the risk model was validated in the Gene Expression Omnibus dataset. Kaplan-Meier curve analysis revealed that CRC patients in the low-risk subgroup had a better survival outcome than those in the high-risk subgroup in the training set, testing set, and overall set. Multivariate analysis demonstrated that the sialylation-related lncRNA signature was an independent prognostic factor for overall survival, progression-free survival, and disease-specific survival prediction. The sialylation lncRNA signature-based nomogram exhibited a robust prognostic performance. Furthermore, enrichment analysis showed that cancer hallmarks and oncogenic signaling were enriched in the high-risk group, while inflammatory responses and immune-related pathways were enriched in the low-risk group. The comprehensive analysis suggested that low-risk patients had higher activity of immune response pathways, greater immune cell infiltration, and higher expression of immune stimulators. In addition, we determined the sialylation level in normal colonic cells and CRC cell lines by flow cytometry combined with immunofluorescence, and verified the expression levels of seven lncRNAs using real-time quantitative polymerase chain reaction. Finally, combined drug sensitivity analysis using the Genomics of Drug Sensitivity in Cancer, Cancer Therapeutics Response Portal, and Profiling Relative Inhibition Simultaneously in Mixtures indicated that the sialylation-related lncRNA signature could serve as a potential predictor for chemosensitivity. Collectively, this is the first sialylation lncRNA-based signature for predicting the prognosis, immune landscape, and chemotherapeutic response in CRC, and may provide vital guidance to facilitate risk stratification and optimize individualized therapy for CRC patients.

## Introduction

Colorectal cancer (CRC) was the third most common and second most deadly tumor in 2020 worldwide (1). The 5-year survival rate for patients with metastatic CRC is only 14%, which highlights the need for early and precise recognition of different risk groups of patients for proper and personalized treatment. The normal staging process follows the criteria of the American Joint Committee on Cancer (AJCC) (2), which is the TNM staging system based on tumor size (T), node involvement (N), and presence of metastasis (M). The consensus molecular subtypes (CMSs) (3) of CRC have also been proposed as a gene expression-based subtyping. Although these two staging strategies are valuable in CRC treatment selection, patients within the same subtype can still exhibit a wide variation of clinical outcomes; thus, more refined classification methods are needed to provide a more personalized therapeutic strategy. Additionally, resistance of cancer cells to chemotherapy and targeted drugs remains common and significantly hinders the management of cancer (4). Tools to predict the drug sensitivity of each patient can be helpful to guide treatment decisions. More recently, treatment with antibodies to programmed cell death protein 1 (PD-1), one of the immunological approaches, has been shown to be successful only in treating microsatellite instability-high tumors, which leaves 85% of CRC patients unable to benefit from immunotherapy (5–8). New versions of immune checkpoint inhibitors are expected to expand the efficacy of immunotherapy. Hence, the discovery of novel biomarkers or therapeutic targets to predict the prognosis, response to treatment, and development of CRC is urgently required.

Sialylation is a posttranslational modification and is controlled by families of sialyltransferases, transporters, and neuraminidases. This process plays a pivotal biological role in the maintenance of cell-cell interactions and is involved in many pathological conditions such as cancer, embryonic lethality, and immune system abnormality (9). Mounting evidence indicates that aberrant sialylation is very common in human cancers. It has been shown to participate in oncogenesis, and contribute to tumor cell dissociation and invasion, immune evasion, and resistance to therapy (10). Thus, sialyltransferases have recently been proposed as a potential target for anticancer treatment (11). Sialic acid-binding immunoglobulin-like lectins (Siglecs), such as Siglec-5, -7, -9, and -10, have been praised as the next immune checkpoint targets to boost antitumor immune responses because they are extensively distributed on various tumor-infiltrating cells, including a subset of T cells (12, 13), neutrophils (14), and natural killer cells. For example, Siglec-10 on tumor-associated macrophages interacted with tumor-expressed CD24, and blockage of both molecules led to a reduction in tumor growth and an increase in survival time (15). In addition, Siglec-15 was recently recognized as an immune suppressive receptor in cancer, and found to be exclusively expressed with programmed cell death ligand 1 (PD-L1) (16, 17); this implies that it can be an alternative target for non-responders to PD-1 therapy. Currently, the phase I/II trial of a Siglec-15 antibody (NC318) is ongoing. In addition, hypersialylation has been considered essential for cancer growth and progression as tumors imitate host-like cell-surface sialylation. Research on sialyltransferases has generally focused on carcinomas. For example, ST6 beta-galactoside α-2,6-sialyltransferase 1 (ST6GAL1), one of the sialytransferases that catalyze the α-2,6-sialylation of *N*-acetyllactosamine, was associated with increased metastasis and poor survival in CRC (18, 19). Synthesized α-2,6-sialylation glycan ligands showed high specificity for Siglec-2, a crucial target on B cells for immunotherapy (20, 21). Therefore, biomarkers of sialylation-related molecules might be valuable for predicting survival outcomes in patients with CRC.

Long noncoding RNAs (lncRNAs) are non-protein coding transcripts longer than 200 nucleotides. Their annotation has been improved with the development of sequencing technology (22). lncRNAs play a significant role in the development and progression of cancer through epigenetic modifications or translational regulation. Although there have been thorough mass spectrometry studies on aberrant sialylation (23), there is still very little research on sialylation-related lncRNAs in cancer. ST8SIA6 antisense RNA 1 (*ST8SIA6-AS1*) (24), long intergenic non-protein coding RNA 1296 (*LINC01296*) (25), maternally expressed gene 3 (*MEG3*) (26), and ST3Gal6 antisense RNA 1 (*ST3Gal6-AS1*) (27) are lncRNAs that have been examined in relation to cancer progression. Additionally, Quan Du discovered *ST6GAL1*-related competing endogenous RNA networks in alcohol-related esophageal cancer (28). Nevertheless, it is still ambiguous whether sialylation-related lncRNAs could be useful in predicting CRC patient prognoses or treatment responses. Considering the large number of unknown lncRNAs, we believe that a number of potential lncRNAs take part in sialylation and Siglec interactions, and whether they have clinical significance in CRC should be explored. Therefore, there is an urgent need to identify sialylation-associated lncRNA biomarkers for predicting the prognosis and response to treatment in patients with CRC.

In this study, we identified prognostic lncRNAs related to sialylation and Siglec function pathways, and we successfully established a sialylation-related lncRNA signature in CRC. We comprehensively investigated the associations of the sialylation-related lncRNA signature with clinicopathological features, underlying mechanisms, somatic mutations, the immune microenvironment, and chemotherapeutic responses in CRC. Our results strongly suggest that our sialylation-related lncRNA signature can serve as a novel biomarker for estimating the risk of mortality and can clarify vital aspects of the signaling pathways and mechanisms of CRC progression. Additionally, it may be beneficial for personalized therapy in patients with CRC.

## Materials and methods

### Samples and data preprocessing

A total of 543 cases of colon adenocarcinoma and rectal adenocarcinoma with complete clinical information were downloaded from The Cancer Genome Atlas (TCGA, https://www.cancer.gov/tcga) and screened for further analysis. The expression data of 51 normal tissue samples were also obtained from the TCGA portal. Human gene symbols were annotated to Ensembl gene IDs by GENCODE V37 (22) through R, where lncRNAs were recognized as a subset of the main annotation file. The fragments per kilobase million (FPKM) of each sequence were downloaded and scaled to a total depth of 10^6^ fragments per sample, which is transcripts per million, to facilitate further analysis. GSE147602 (29) and GSE198103 (https://www.ncbi.nlm.nih.gov/geo/query/acc.cgi?acc=GSE198103) from the Gene Expression Omnibus (GEO) (https://www.ncbi.nlm.nih.gov/geo/) database was used for external validation in this study. The somatic mutation and neoantigen data for CRC were acquired from the TCGA database analyzed by the package “maftools” (30), and CMSs for patients were obtained from the Colorectal Cancer Subtyping Consortium Synapse (3). The median value of the calculated risk score was used as a cutoff point to divide the low- and high-risk groups in the TCGA and GEO datasets.

### Screening sialylation-related lncRNAs and construction of a prognostic model

Genes participating in the biological sialylation process were recognized through the Molecular Signatures Database (MSigDB) (31–33), which contains sialyltransferases, transporters, and neuraminidases (Supplementary Table S1). With a threshold of *P<* 0.001 and an absolute value of correlation coefficient > 0.3, we confirmed 1268 sialylation-related lncRNAs. The patient cohort was randomly divided into training and testing groups in a 70:30 ratio (Supplementary Table S2). After univariate Cox regression, sialylation-related lncRNAs with *P*< 0.01 were retained for the next step. Under a stepwise algorithm and multivariate Cox regression, a sialylation-related lncRNA prognostic signature was constructed to predict overall survival (OS) in CRC patients by computing a risk score. The risk score for each patient was calculated as follows:


, $$Risk\;score=\;\sum_{i=1}^{n}Coefficient\left(lncRNA_{i}\right)*Expression\left(lncRNA_{i}\right)$$

where *Coefficient* is calculated using the multivariate Cox regression model, and *Expression* refers to the expression of lncRNAs in both the TCGA and GEO.

### Validation of the prognostic model and construction of a nomogram

The median risk score was used as the dividing point for labeling the patients as high- or low-risk patients. Kaplan-Meier survival analysis of OS, progression-free survival (PFS), and disease-specific survival (DSS) was performed between the risk groups, and the association between clinical parameters and the risk score was assessed through univariate and multivariate Cox regression. A nomogram was drawn combining the significant independent factors (*P*< 0.05) along with the C-index and receiver operating characteristic (ROC) curve analysis to demonstrate its efficiency in predicting survival outcome of patients at 1, 3, 5, and 7 years.

### Functional enrichment analysis

The R packages “limma” (34) and “egdeR” (35–37) were used to determine differentially expressed genes (DEGs) between the two risk groups, with a threshold of an adjusted *P<* 0.05 and an absolute value of log(fold change (FC)) > 0.5. The Kyoto Encyclopedia of Genes and Genomes (KEGG) pathway and Gene Ontology (GO) analyses were performed using the gene names, and Gene Set Enrichment Analysis (GSEA) was conducted with the gene names and log(FC) between the phenotype labels “high risk” and “low risk.” All these analyses were conducted in the R package “clusterProfiler” (38).

### Analysis of immune cell infiltration

Tumor Immune Estimation Resource (TIMER, https://cistrome.shinyapps.io/timer/) (39, 40) is a comprehensive resource to analyze immune infiltration. In this study, we examined the level of immune infiltration in the high- and low-risk groups using TIMER, Cell-type Identification By Estimating Relative Subsets of RNA Transcripts (CIBERSORT) (41), the Microenvironment Cell Populations-counter (MCP-counter) (42), and Estimating the Proportion of Immune and Cancer cells (EPIC) (43) methods. We used gene set variation analysis (GSVA) to calculate the immune cell infiltration in each patient as well.

### Drug sensitivity prediction

We downloaded drug sensitivity data from three databases, Genomics of Drug Sensitivity in Cancer (GDSC, https://www.cancerrxgene.org/) (44), The Cancer Therapeutics Response Portal (CTRP, https://portals.broadinstitute.org/ctrp.v2.1/) (45–47), and Profiling Relative Inhibition Simultaneously in Mixtures (PRISM, https://www.theprismlab.org/) (48). The half-maximal inhibitory concentration (IC50) in GDSC and area under the dose-response curve (AUC) in CTRP and PRISM negatively correlated with the drug sensitivity. We also acquired the transcription data of cell lines from DepMap Public 22Q2 (https://depmap.org/portal/download/), and the prediction was conducted by using the R package “Oncopredict” (49).

### Cell culture

Human primary colonic epithelial cells (HCoEpiC) and colonic cancer cells, including LoVo and HCT116, were purchased from BNBIO Company (Beijing, China). HCoEpiC and HCT116 cells were cultivated in F12/Dulbecco’s modified Eagle medium (Gibco, Invitrogen, Paisley, UK), and LoVo cells were grown in F-12K medium (Gibco, Invitrogen, Paisley, UK). Medium was supplemented with 10% fetal bovine serum (Corning, NY, USA) and 1% penicillin-streptomycin (10,000 units/mL penicillin, 10,000 μg/mL streptomycin; Gibco, Invitrogen, Paisley, UK). All the cells were cultured at 37°C in a humidified incubator containing 5% CO_2_.

### Flow cytometry

Cells were harvested by trypsin/EDTA (Gibco, Invitrogen, Paisley, UK) treatment for 2 min at 37°C. After adding culture medium and centrifugation to terminate the digestion, phosphate-buffered saline (PBS; Solarbio, Beijing, China) was added and cells were centrifuged twice. Cells were then resuspended in 100 μL of PBS (2x10^6^ cells/100 μL) and mixed with 100 μL fluorescein-conjugated *Sambucus nigra* lectin (FITC-SNA; Vector Labs; L32479; 20 μg/mL) for 30 minutes at 4°C in the dark. After washing with PBS, filtering, and centrifuging, the supernatant was discarded, and stained cells were analyzed by Image-Stream MarkII imaging flow cytometry. The results were analyzed by IDEAS statistical image analysis software (Amnis, EMD-Millipore, Seattle, WA, USA).

### Immunofluorescence

After growing overnight on 35-mm glass bottom dishes (Cellwis, Mountain View, CA), cells were washed twice with PBS and incubated with a blocking solution (Vector Labs; SP-5040-125) at 4°C for 15 minutes to avoid background staining or false positive results. FITC-SNA diluted in blocking solution (20 μg/mL) was then added at 4°C for 30 minutes. Cells were washed twice with PBS, and fixed with 2% paraformaldehyde (Macklin, P804536) for 30 min at room temperature. Cells were mounted on slides with antifade mounting medium containing 4′,6-diamidino-2-phenylindole (DAPI, Solarbio, Beijing, China), to stain the nuclei at the same time. Samples were observed with a confocal laser scanning microscope (Olympus FV3000, Tokyo, Japan).

### Real-time quantitative PCR validation

We extracted the total RNA from HCoEpiC, LoVo, and HCT116 cells using an RNeasy kit (Beyotime, Shanghai, China, R0027) in accordance with the manufacturer’s instructions. Then, we reverse-transcribed 1 μg of total RNA using SuperScript II reverse transcriptase (TaKaRa, Japan, RR047). Quantitative PCR analysis was performed using SYBR Green Master Mix (TaKaRa, Japan, RR820) with an ABI 7900 HT real-time PCR system. The primer sequences for RT-qPCR are listed in Supplementary Table S3.

### Statistical analysis

R software (version 3.2), SPSS 25.0 (IBM Corp., Armonk, New York, USA), and GraphPad Prism 8.0 (GraphPad Software Inc., San Diego, CA, USA) were used for data processing and visualization. Continuous variables were analyzed using the Wilcoxon rank-sum test or Kruskal-Wallis test, and categorical variables were analyzed by Fisher’s exact test or chi-square test (χ^2^). Pearson correlation and Spearman’s correlation analyses were used to assess the relationship between mRNA and lncRNA, and between the risk score and expression of genes. The significance of large-scale multiple tests was evaluated by the Benjamini-Hochberg method.

## Results

### Recognition of a prognostic sialylation-related lncRNA signature

**Figure 1:**
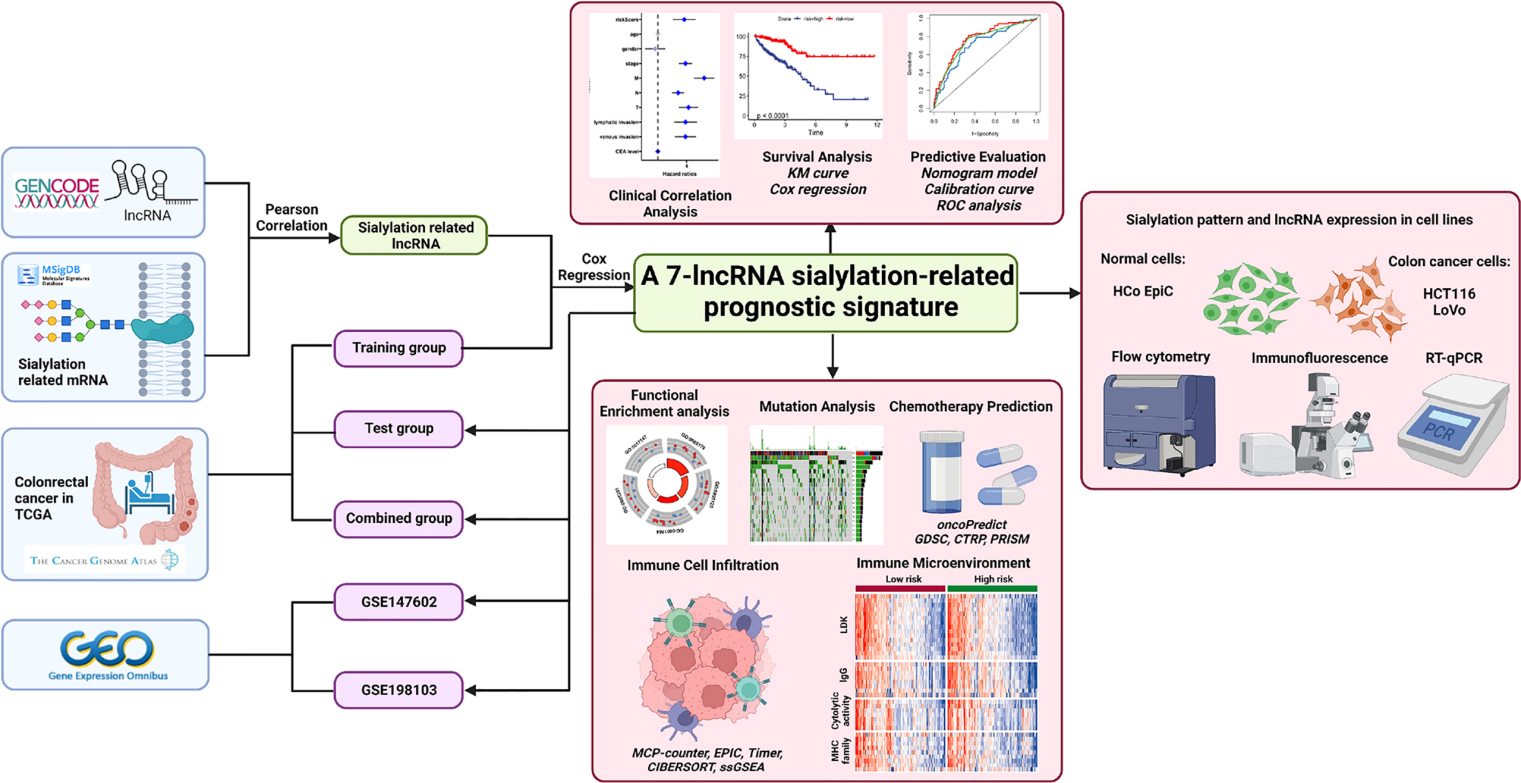
Flowchart of this study.

**Figure 2:**
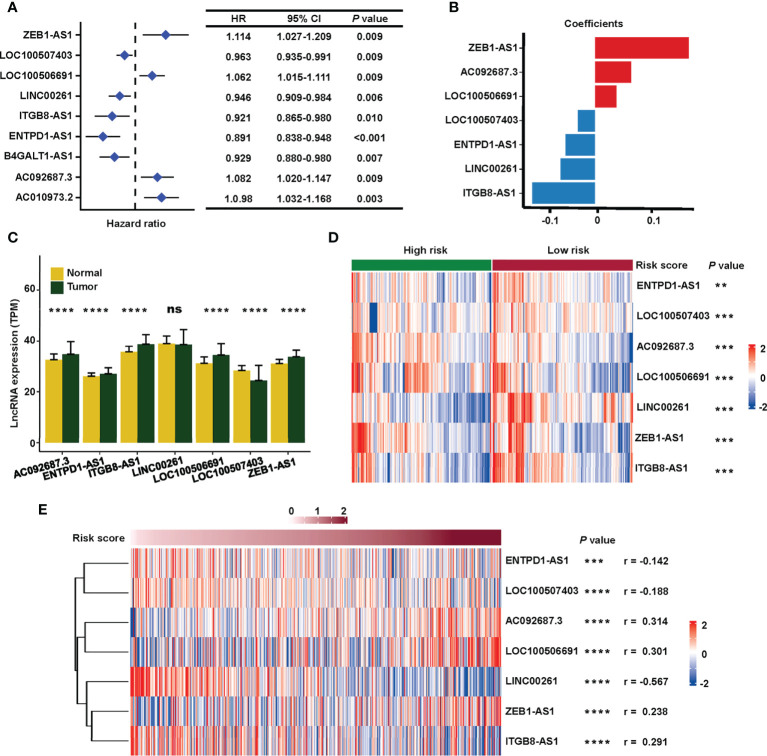
Construction of the sialylation-related lncRNA signature. **(A)** HR and CI of nine lncRNAs with *P*< 0.001 in a univariate Cox regression for OS. **(B)** Coefficients of the seven lncRNAs in the prognosis signature. **(C)** Expression of the lncRNAs in normal (n = 51) and cancer (n = 543) tissues in the TCGA dataset. **(D)** Expression profiles of the lncRNAs in high- and low-risk patients in the combined cohort. **(E)** Correlation of the expression of seven sialylation-related lncRNAs with risk scores by the Spearman test. HR, hazard ratio; CI, confidence interval; and OS, overall survival. ****P< 0.0001; ***P< 0.001; **P< 0.01; *P< 0.05.

The process for exploring the prognostic value of sialylation-related lncRNA expression in colorectal cancer is shown in **Figure 1**. First, 543 patients with complete clinical information were randomly divided into the training and validation sets. We downloaded 120 mRNAs involved in sialic acid and Siglec interaction from the MsigDB website with lncRNA annotations. A total of 1268 lncRNAs strongly correlated with the mRNAs as revealed by the Pearson correlation with a threshold of |r| > 0.3 and *P*< 0.001 in the training group. We next focused on these sialylation-related lncRNAs; univariate Cox regression analysis showed nine lncRNAs that were significantly associated with patient OS (*P*< 0.01, **Figure 2A**). ZEB1 antisense RNA 1 (*ZEB1-AS1*), *LOC100506691*, *AC092687.3*, and *AC010973.2* had a hazard ratio (HR) of more than one, which indicated that they were risk factors. The remaining five lncRNAs, *LOC100507403*, *LINC00261*, ITGB8 antisense RNA 1 (*ITGB8-AS1*), ENTPD1 antisense RNA 1 (*ENTPD1-AS1*), and B4GALT1 antisense RNA 1 (*B4GALT1-AS1*), were protective factors with HR< 1.

Next, a signature based on the seven sialylation-related lncRNAs was constructed by multivariate Cox regression and a stepwise algorithm. The genes were as follows: *ZEB1-AS1*, *AC092687.3*, *LOC100506691*, *LOC100507403*, *ENTPD1-AS1*, *LINC00261*, and *ITGB8-AS1*. The coefficients of each lncRNA (**Figure 2B**) had the same trend as their HR value in the univariate Cox regression. Specifically, *ZEB1-AS1*, *AC092687.3*, and *LOC100506691* showed positive coefficients leading to a higher risk score, while *LOC100507403*, *ENTPD1-AS1*, *LINC00261*, and *ITGB8-AS1* had negative coefficients as they were protective factors.

The expression level of five of the lncRNAs was significantly higher in tumor tissue, except for *LINC00261* (with no difference) and *LOC100507403* (which was significantly higher in normal tissue) (**Figure 2C**). In the combined set, seven sialylation-related lncRNAs were significantly differentially expressed between the high- and low-risk groups, as defined by the median risk score (**Figure 2D**). Additionally, the relationship of the risk score with the seven sialylation-related lncRNAs was calculated by the Spearman test; *LINC00261* showed the highest negative correlation (r = −0.567, **Figure 2E**).

### Validation of the signature based on the seven sialylation-related lncRNAs for survival prediction and as an independent prognostic factor

**Figure 3:**
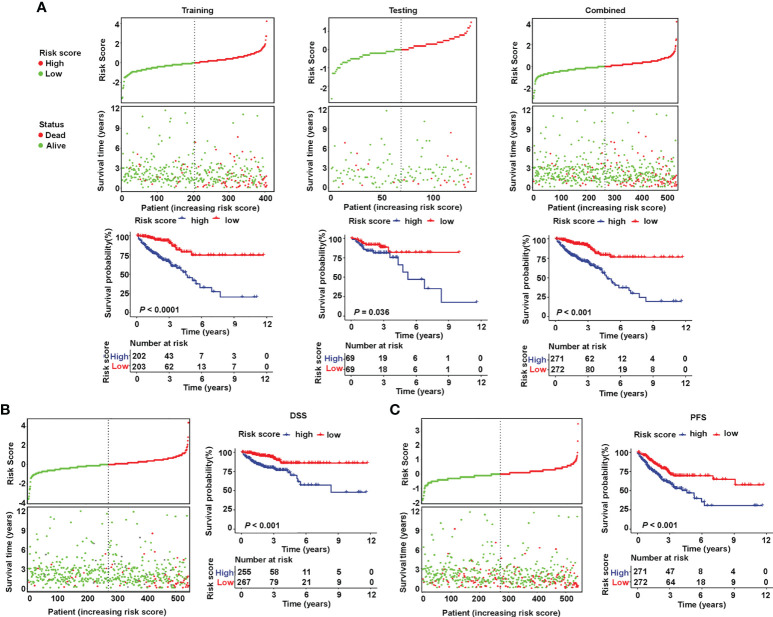
Validation of the prognostic value of the sialylation-related lncRNA signature in TCGA. **(A)** Distribution of risk score (high or low) and status (dead or alive) and KM curves of OS in the training (left), testing (medium), and combined (right) sets. **(B, C)** Distribution of risk score and status and KM curves of DSS and PFS in the combined set. KM, Kaplan-Meier survival test; OS, overall survival; DSS, disease-specific survival; and PFS, progression-free survival.

The patients were divided into a high- or low-risk type according to the median of the calculated risk score of each set. The risk score and the survival status of the training, testing, and combined sets are displayed in **Figure 3A** (top). Higher mortality was observed in the high-risk group, indicating a worse prognosis both in the training set (**Figure 3A** (left), *P<* 0.001) and in the testing set (**Figure 3A** (middle), *P* = 0.036). High-risk patients had a significantly worse OS (**Figure 3A** (right), *P<* 0.001), DSS (**Figure 3B**, *P*< 0.001), and PFS (**Figure 3C**, *P*< 0.001) in the TCGA combined set.

**Figure 4:**
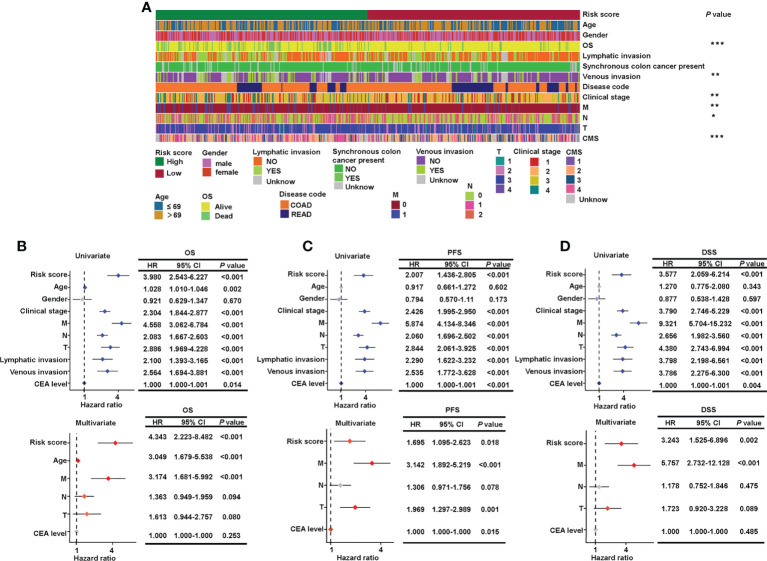
Verification of the relationship between risk score and clinicopathological features. **(A)** Clinical details of 543 patients and the significant difference between high- and low-risk groups. Forest plots of the hazard ratios predicting OS **(B)**, PFS **(C)**, and DSS **(D)** in univariate and multivariate Cox regression models. OS, overall survival; DSS, disease-specific survival; and PFS, progression-free survival. ***P< 0.001; **P< 0.01; *P< 0.05.

To examine the relationship between clinicopathological features and risk score, a χ^2^ test and Cox regression were used on the combined set. The high- and low-risk groups differed in OS, venous invasion, clinical stage, metastatic stage, node stage, and CMS as showed by the χ^2^ test (**Figure 4A**). Furthermore, the risk groups stratified by the signature based on the seven sialylation-related lncRNAs were significantly independent of the M, N, and T stages, and carcinoembryonic antigen level, and this finding was confirmed by the multiple Cox regression results of OS (HR = 4.343, 95% CI = 2.223–8.482, *P*< 0.001, **Figure 4B**), PFS (HR = 1.695, 95% CI = 1.095–2.623, *P* = 0.018, **Figure 4C**), and DSS (HR = 3.243, 95% CI = 1.525–6.896, *P* = 0.002, **Figure 4D**). These results implied that our sialylation-related lncRNA signature for CRC is reliable for predicting survival outcomes and may serve as an independent prognostic factor.

### Construction and validation of a nomogram combining clinical characteristics

**Figure 5:**
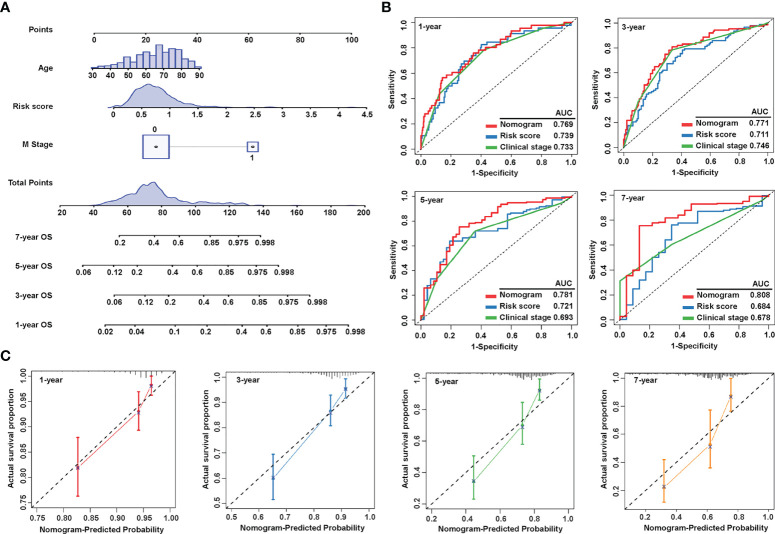
Construction and evaluation of the nomogram. **(A)** Nomogram constructed by three independent prognostic factors (risk score, metastatic **(M)** stage, and age) predicts 1-, 3-, 5-, and 7-year overall survival. **(B)** ROC curves show the predictive accuracy of the nomogram, risk score, and the clinical stage. **(C)** Calibration curves for 1-, 3-, 5-, and 7-year survival. ROC, receiver operating characteristic; and AUC, area under the curve.

As the risk score was independent of other clinical characteristics, we attempted to optimize the signature for more clinically efficient utilization. Based on the multivariate Cox regression analysis of OS, we integrated age, M stage, and risk score to generate a nomogram predicting the probability of survival after 1, 3, 5, and 7 years (**Figure 5A**). ROC curves to assess the nomogram’s prediction accuracy showed that the nomogram performed better than clinical stage and risk score at 1 year (AUC = 0.769), 3 years (AUC = 0.771), 5 years (AUC = 0.781), and 7 years (AUC = 0.808) (**Figure 5B**). Additionally, calibration curves were drawn to examine the consistency of the actual measured outcome and predicted prognostic value. The results showed that the predicted outcome of the nomogram was highly consistent with the observed one (**Figure 5C**). These data showed that we successfully established a nomogram that combined a sialylation-related lncRNA signature and clinical characteristics for a more accurate prediction.

### Potential mechanism analysis of the sialylation-related lncRNA signature

**Figure 6:**
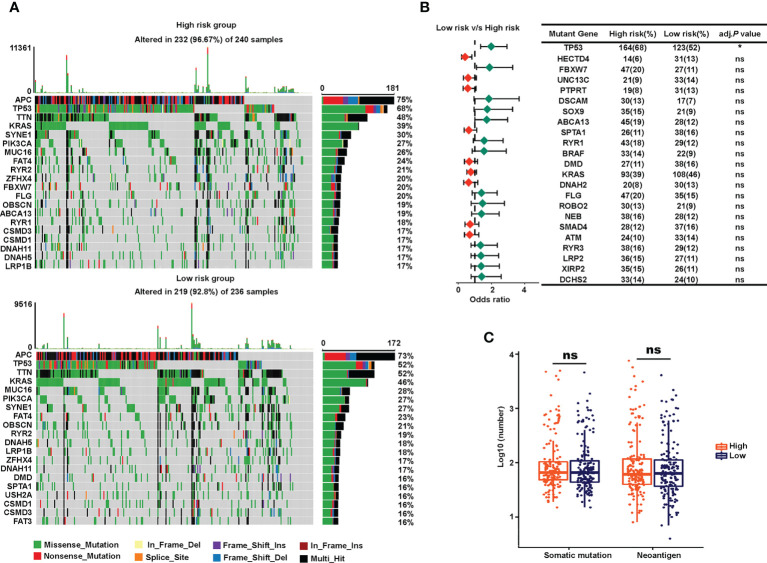
Somatic mutations of the high- and low-risk groups. **(A)** Waterfall of the top 20 tumor somatic mutations in the high-risk (up) and the low-risk (down) cohorts. **(B)** Forest plot showing the odds ratios of the 20 most significantly differentially distributed mutated genes between the two risk groups. **(C)** Numbers of somatic mutations and neoantigens in the high- and low-risk TCGA groups. *P< 0.05; ns, not significant.

To explore the underlying mechanism by which the risk signature stratified the prognosis of patients, we performed mutation, KEGG pathway, GSEA, and GO analyses. Since somatic mutations are the essential cause of malignancy (50), we visualized the landscape of mutation profiles in TCGA using the “maftool” package, where the top 20 most frequent mutations were displayed separately in the high- and low-risk groups (**Figure 6A**). A Fisher test showed that there were no significant differences in the ratio of mutation occurrences between the two risk groups, except for mutated gene TP53 (**Figure 6B**). In addition, the number of somatic mutations and neoantigens showed no significant differences between the two groups (**Figure 6C**). These results indicate that the prognostic significance of the sialylation lncRNAs-based signature may be independent of somatic mutations.

**Figure 7:**
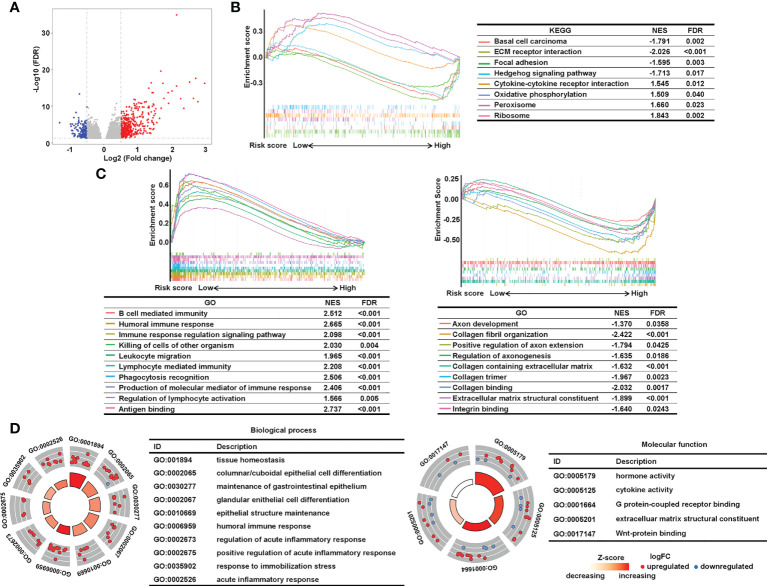
Analysis of underlying biological pathways of the sialylation-related lncRNA signature. **(A)** Volcano plot of differentially expressed genes in the high- and low-risk cohorts with a threshold of FDR > 0.05 and absolute log2(FC) > 0.5. Upregulated pathways enriched in the low-risk group (NES > 1) and the high-risk group (NES< 1) of KEGG **(B)** and GO **(C)** with FDR< 0.05. **(D)** GO enrichment of differentially expressed genes. FC, fold change; FDR, false discovery rate; and NES, normalized enrichment score.

Next, we conducted pathway analysis and GSEA to investigate the underlying biological importance of the sialylation-related lncRNA signature. A total of 603 DEGs were identified with their |log_2_(FC)| > 0.5 and adjusted *P*< 0.05 between the two risk groups in the combined set using the R package “edgeR” (**Figure 7A**). GSEA showed that pathways related to the extracellular matrix (ECM), such as basal cell carcinoma, ECM-receptor interaction, focal adhesion, and the hedgehog signaling pathway, were enriched in the high-risk group, and patients with a lower risk score had enriched pathways for cytokine-cytokine receptor interactions, oxidative phosphorylation, peroxisomes, and ribosomes (**Figure 7B**). Furthermore, GSEA showed that immune response pathways, such as B cell-mediated immunity and immune response regulation, were enriched in the low-risk group (**Figure 7C**). In patients with a higher risk prediction, pathways of collagen and axons were enriched, such as axon development, extracellular matrix structural constituent, collagen fibril organization, and regulation of axonogenesis. We also conducted GO analysis on the 603 DEGs and confirmed that immune processes participated in the sialylation signature-related division of CRC patients (**Figure 7D**). The pathway analysis illustrated that immunological and extracellular composition may explain the discriminatory power of our signature.

### Immune microenvironment of the sialylation-related signature score

**Figure 8:**
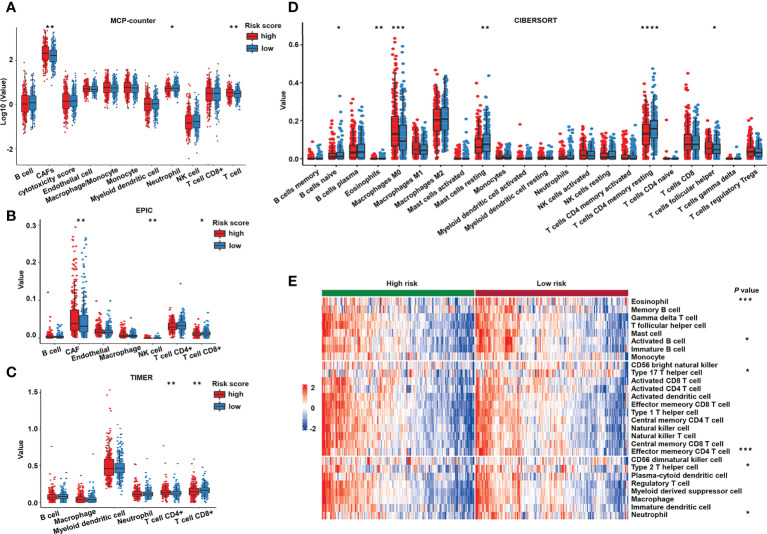
Immune cell infiltration of the different risk groups. Estimated immune cell infiltration value in different risk groups by MCP-counter **(A)**, EPIC **(B)**, TIMER **(C)**, and CIBERSORT **(D)**. **(E)** Heat map of prediction of immune cells infiltration in the high- and low-risk groups by GSVA. GSVA, gene set variation analysis. ****P< 0.0001; ***P< 0.001; **P< 0.01; *P< 0.05.

To further investigate whether the immune process closely correlated with the risk score, we mined TCGA transcriptomic data to explore the difference in tumor immune infiltration between the two risk groups by using deconvolution algorithms in TIMER. Both MCP-counter (**Figure 8A**) and EPIC (**Figure 8B**) showed that cancer-associated fibroblasts, known as critical components of the tumor mesenchyme (51), were significantly higher in patients with a high risk. Antitumor immune cells, such as CD8+ T cells identified by EPIC (**Figure 8B**) and TIMER (**Figure 8C**), and CD4 memory resting T cells and naive B cells identified by CIBERSORT (**Figure 8D**) exhibited higher proportions in the low-risk group. To further validate these findings, we used GSVA to calculate immune infiltration (**Figure 8E**). Interestingly, the proportions of immune cells were higher in the low-risk group compared to the high-risk group, regardless of whether they were antitumor, intermediate, or pro-tumor immune cells, as shown in the GSVA heatmap.

**Figure 9:**
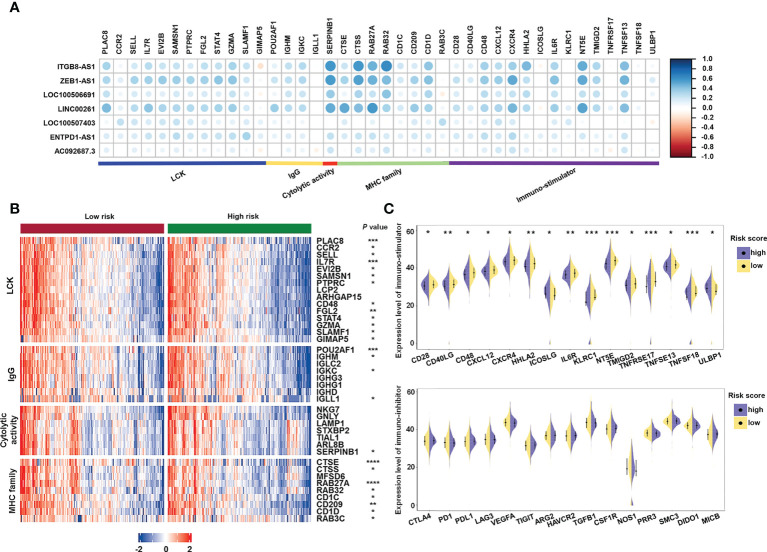
Analysis of the correlation between immune-related genes and sialylation-related lncRNA signature. **(A)** Heatmap of the correlation between expression of immune-related molecules and sialylation-related lncRNAs. **(B)** Expression of genes involved in the LCK pathway, IgG, cytolytic activity, and the MHC family in different risk groups. **(C)** Analysis of immune-stimulator and immune-inhibitor expression with different risk scores. *LCK*, lymphocyte-specific protein tyrosine kinase; IgG, immunoglobulin G; and MHC, major histocompatibility complex. ****P< 0.0001; ***P< 0.001; **P< 0.01; *P< 0.05.

Furthermore, to investigate the immune system metagene, we examined crucial genes involved in inflammatory activities and immune molecules (52). Correlation between the chosen genes and sialylation-related lncRNAs were examined **(** **Figure 9A** **)**. Serpin family B member 1 (*SERPINB1*) and Cathepsin S (*CTSS*) were significantly correlated to four of the seven lncRNAs, *ITGB8-AS1*, *LINC00261*, *ZEB1-AS1*, and *LOC100506691*. Major histocompatibility complex (MHC) genes, such as RAS oncogene family member 32 (*RAB32*), *CTSS*, RAS oncogene family member 27A (*RAB27A*), and cathepsin E (*CTSE*) were strongly correlated with *LINC00261* **(** **Supplementary Figure 1** **)**. Four immune stimulators, which were Interleukin 6 receptor (*IL6R*), 5’-nucleotidase ecto (*NT5E*), C-X-C motif chemokine receptor 4 (*CXCR4*), tumor necrosis factor superfamily member 13 (*TNFSF13*), were all strongly correlated with *ZEB1-AS1* **(** **Supplementary Figure 2** **)**. Specific genes and families, including the lymphocyte-specific protein tyrosine kinase (*LCK*) pathway; immunoglobulin G (IgG), MHC family, and cytolytic activity genes, such as interleukin 7 receptor (*IL7R*), *CD48*, immunoglobulin heavy constant gamma 1 (*IGHG1*), *CTSE*, and *CD209* (**Figure 9B**); and immune stimulators, such as *CD28*, *IL6R*, and *TNFSF13* (**Figure 9C**), had significantly lower expression in the high-risk samples. These data demonstrated that immunogenicity varied between the different risk groups.

### Validation of the sialylation-related lncRNA signature

**Figure 10:**
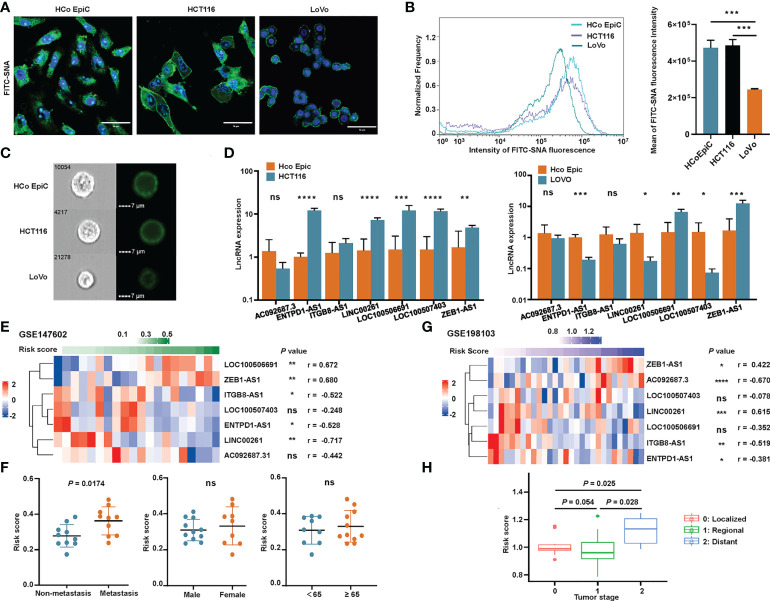
Validation of the sialylation-related lncRNA signature. **(A)** Representative confocal images of normal human colonic epithelial cells (HCoEpiC) and colonic cancer cells including HCT116 and LoVo stained with FITC-SNA. **(B)** Flow cytometry histogram and boxplot showing the α2’-6’-sialylation on the surface of the three cell types. **(C)** Representative images of the three cell types photographed by Image-Stream MarkII. **(D)** Comparison of the seven lncRNAs between HCoEpiC, HCT116 cells, and LoVo cells. **(E)** Expression profile of the lncRNAs in GSE147602. **(F)** Risk score distribution in GSE147602 with different clinical features. **(G)** Expression profile of the lncRNAs in GSE198103. **(H)** Risk score of patients with different tumor stages in GSE198103. ****P< 0.0001; ***P< 0.001; **P< 0.01; *P< 0.05; and ns, not significantly.

We used flow cytometry and immunofluorescence to explore the sialylation pattern on different cell surfaces. We observed that HCoEpiC and HCT116 cells had significantly stronger fluorescence intensities than LoVo cells (**Figures 10A–C** **)** when the cell surface was stained with SNA, a specific lectin that binds to α-2,6 linked sialic acid. These results showed that LoVo cells had a significantly lower sialylation level. To confirm the expression of these seven sialylation-related lncRNA signature genes, RT-qPCR was performed on HCoEpiC and the colonic cancer cells (HCT116 and LoVo). Comparing HCT116 cells with HCoEpiC, we found that *ENTPD1-AS1*, *ITGB81-AS1*, *LINC00261*, *LOC100506691*, *LOC100507403*, and *ZEB1-AS1* were significantly overexpressed in the tumor cells (**Figure 10D**). Comparing LoVo cells with the normal colonic epithelial cells, we found that *LOC100506691* and *ZEB1-AS1* had higher expression in the tumor cells, while *ENTPD1-AS1*, *LINC00261*, and *LOC100507403* had higher expression in the normal cells. *AC092687.3* and *ITGB8-AS1* showed no significant differences between the cell types.

We also used two GEO cohorts, GSE147602 and GSE198103, to investigate the prognostic ability of our signature. In GSE147602, the risk score calculated by the formula showed a strong association with *LOC100506691*, *ZEB1-AS1*, *ITGB8-AS1*, *ENTPD1-AS1*, and *LINC00261* (**Figure 10E**). As shown in **Figure 10F**, the risk score significantly correlated with metastasis stage but was not significantly associated with other clinicopathological features such as gender and age. In GSE198103, the risk score was strongly correlated with *AC092687.3*, *LINC00261*, and *ITGB8-AS1* (**Figure 10G**). The risk score of patients with a distant metastasis stage was significantly higher than ones with a localized or regional stage (**Figure 10H**). The ability of the sialylation-related lncRNA signature to evaluate the prognosis of CRC patients was clearly validated by these two cohorts.

### Drug sensitivity prediction in CRC patients in the high- and low-risk groups

**Figure 11:**
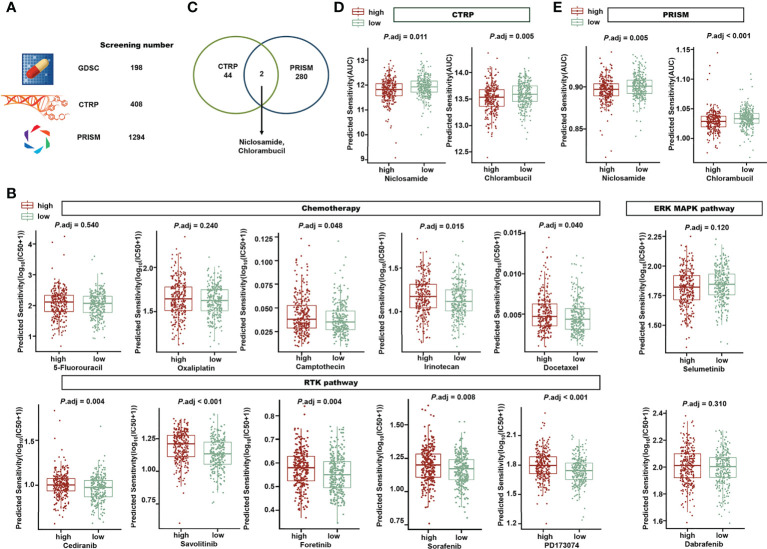
Exploration of the risk signature and drug sensitivity. **(A)** A total of 1900 compounds from GDSC, CTRP, and PRISM were screened to investigate promising drugs for clinical treatment. **(B)** In the high- or low-risk groups, different predicted IC50 values of drugs in chemotherapy, the RTK pathway, and the ERK/MAPK pathway are displayed. **(C)** Venn diagram showing the two candidate compounds between CTRP and PRISM. Niclosamide and chlorambucil are the two compounds predicted with significantly lower AUCs in the high-risk group (*P* adj< 0.05) in CTRP **(D)** and PRISM **(E)**. GDSC, Genomics of Drug Sensitivity in Cancer; CTRP, The Cancer Therapeutics Response Portal; PRISM, Profiling Relative Inhibition Simultaneously in Mixtures; RTK, receptor tyrosine kinase; and ERK/MAPK, extracellular signal-regulated kinase/mitogen-activated protein kinase.

To evaluate the application of our sialylation-related signature in clinical therapy and identify promising drugs for high-risk patients, we analyzed the chemotherapeutic drug sensitivity. After predicting the sensitivity of 1900 compounds for 543 patients, we conducted a Wilcoxon test between the two risk groups and set a threshold of an adjusted *P*< 0.05 (**Figure 11A**). In 198 compounds from the GDSC, there were 49 compounds displaying a significantly low IC50 in the low-risk group, compared with only five in the high-risk group. Drugs commonly used in clinical chemotherapy are shown in **Figure 11B**. The IC50 of 5-fluorouracil and oxaliplatin did not differ between the risk groups, while the IC50 of camptothecin, irinotecan, and docetaxel were significantly lower in the low-risk group, which implied that patients with a lower risk score could be more sensitive to these chemotherapy compounds. Compounds targeting the receptor tyrosine kinase (RTK) pathway, such as cediranib, savolitinib, foretinib, and sorafenib, were predicted to be more effective in the low-risk group. There were no differences between the two risk groups in compounds targeting the extracellular signal-regulated kinase/mitogen-activated protein kinase (ERK/MAPK) pathway.

Forty-four compounds in the CTRP and 280 compounds in PRISM were identified with a significantly lower AUC in the high-risk group. Niclosamide and chlorambucil were at the intersection of the promising complexes of the two databases (**Figure 11C**), which means that they were both predicted with a lower AUC in the high-risk group in the CTRP (**Figure 11D**) and PRISM (**Figure 11E**). Our findings suggest that the risk signature can support clinical drug selection, and niclosamide and chlorambucil may be appropriate for high-risk patients.

## Discussion

The overexpression of sialic acid, hypersialylation, has been observed to stimulate tumor deterioration and dampen the immune response (21) with the involvement of Siglecs. Siglecs are commonly expressed on NK and tumor-infiltrating T cells and are involved in regulating immune cells. To normalize the immune system, molecules that block Siglecs, such as antibodies against Siglec-15 (16, 17), currently under a clinical safety and tolerability trial, are considered promising in advanced solid tumors. The roles of lncRNAs, a type of RNA that meditates multiple pathways and has a promising future in early cancer diagnosis, remain unclear in sialylation and Siglec interactions. Given the previous evidence that multiple gene-related signatures based on specific biological functions were superior in predicting the prognosis and individualized therapy in patients with CRC (53–56), in the present study, we aimed at exploring the clinical significance of a sialylation lncRNAs-based signature.

In this study, we developed a novel signature based on seven lncRNAs, all of which have a strong correlation with the sialic acid and Siglec pathways. Three of them (*LOC100507403*, *ENTPD1-AS1*, and *AC092687.3*) remain functionally uncharacterized. *ZEB1-AS1* has been reported as an oncogene in prostate cancer (57), and was found to activate *ZEB1* epigenetically. *ZEB1-AS1* was also reported upregulated in colorectal cancer tissues (58). Interestingly, antiproliferative effects of metformin in gastric cancer have been shown to lead to suppression of *LOC100506691* (59). According to Xiaoting Lin (60), *ITGB8-AS1* regulated cell proliferation and tumor growth of CRC by regulating focal adhesion signaling, which indicates a therapeutic potential. Dysregulation of *LINC00261* has been found to play vital roles in pancreatic cancer (61–63), gastric cancer (64), hepatocellular carcinoma (65–67), and colon cancer (68). Evidence of the role of sialic acid-related lncRNAs in cancer is sparse, though promising, and integrating these seven sialic acid-related lncRNAs into a 7-lncRNA signature exhibits novel clinical significance. Our results show that this 7-lncRNA signature can distinguish the prognosis of patients and theoretically support clinical treatment strategies. Additionally, a nomogram containing age, M stage, and risk score was constructed and shown to exhibit a greater predictive ability than the clinical stage, which implies an optimization of the current clinical classification for staging. Moreover, the risk score calculated by the 7-lncRNA signature was independent of the main prognostic factors in CRC. To exhibit the prognostic ability of the signature, we chose the median value rather than the optimal cutoff values to divide the patients into high- and low-risk groups for the following reasons. First, the median value is commonly considered robust as an unsupervised approach. The application of the median risk score as a cutoff can be easily found in studies constructing both mRNA (69) and lncRNA (70, 71) signatures. Second, the optimal cutoff values may show excellent performance in one specific cohort but lack universality. The application of the median value as a cutoff can be applied widely and easily with objectivity.

We assume that the development of fibroblasts and stronger immune activity may result in better outcomes in the low-risk group. As tumor stroma consists of immune cells, activated fibroblasts, ECM (72), and other basement membrane constituents (73), the progression of the tumor stroma stimulates the progression and metastasis of cancer. In high-risk patients, cancer-associated fibroblasts, as producers of pro-tumorigenic signals (51, 74–76), were predicted to show a higher infiltration rate by Epic and MCP-counter. In addition, ECM-receptor interactions, collagen interactions, hedgehog signaling, and focal adhesions, pathways that can be classified as fibroblast-related, were enriched. This suggests that our signature can indicate epigenetic changes, and that these seven sialylation-related lncRNAs may participate in the deterioration process of the stroma, which augments tumor growth.

The results of biological pathway and functional enrichment analyses also revealed that pathways concerning immune responses, such as humoral immune responses, leukocyte migration, phagocytosis recognition, regulation of lymphocyte activation, and antigen binding, were enriched in the low-risk group. Moreover, the immune cell infiltration estimated by five algorithms indicated that low-risk patients tended to display a higher antitumor immune activity. The immune stimulators were also more highly expressed in the low-risk group; however, inhibitors of the immune response show no difference between the risk groups. The correlation analysis further revealed that several genes in immune biological processes had a strong connection with the 7-lncRNA signature. In particular, genes in the MHC pathway, *RAB27A*, *RAB32*, and *CTSS*, had a correlation higher than 0.4 with *ITGB8-AS1*, *ZEB1-AS1*, and *LINC00261*. *NT5E*, *TNFSF13*, *CXCR4*, and *IL6R*, all related to immune stimulation, were correlated with *ITGB8-AS1*, *ZEB1-AS1*, and *LINC00261*. This implies that *ITGB8-AS1*, *ZEB1-AS1*, and *LINC00261* may play a role in the MHC pathway or immune stimulation by interacting with these genes in some manner. Taken together, patients with low risk exhibited a strong immune response that could be a better prognostic indicator than somatic mutations. Increased sialylation in tumor cells has been found to lead to resistance to chemotherapeutics, such as gefitinib (77) and cisplatin (78, 79). In this study, the risk groups grouped by the sialylation-related lncRNA signature also showed different drug sensitivity; in particular, the low-risk group had better outcomes after receiving drugs regulating the RTK pathway. Niclosamide and chlorambucil were recognized as promising treatments for the high-risk group, given that their predicted AUCs were significantly lower in the high-risk group, which means that a higher efficacy is expected with these drugs. Interestingly, niclosamide has been repurposed as a *STAT3* inhibitor in CRC treatment (80), and *STAT3*, an important therapeutic target, has recently been found to be meditated by sialylated c-Met in CRC (81). Our results linked clinical therapy outcomes with TCGA expression data, and these findings may guide drug selection for patients with different sialylation-related lncRNA signature scores in the future.

In the validation of the seven lncRNAs between the normal human colonic cells (HCoEpiC) and colonic cancer cells, the expression of *LOC100506691* and *ZEB1-AS1* was higher in the cancer cells, which agreed with the comparison of normal and cancer tissues in the TCGA dataset. CRC tissue contains cancer cells and stromal cells, such as immune cells, fibroblasts, and blood vessels, which may explain the inconsistency of other lncRNA expression between our RT-qPCR results and the TCGA database. The difference between two colonic cancer cells, HCT116 and LoVo, also cannot be ignored. Expression of *ZEB1-AS1* was higher in CRC tissue than in paired noncancerous tissue, and higher in colonic cancer cell lines than in human normal cell lines, which was in agreement with our findings (82). Despite the absence of studies on *LOC100506691* in CRC, there is evidence that *LOC100506691* plays an oncogenic role in gastric cancer cell growth (59), which is in accordance with its high expression level in CRC. We believe that *LOC100506691* and *ZEB1-AS1* have promising value in the diagnosis of CRC and the available data provide clues regarding their possible function in tumorigenesis and progression, but more experiments are needed to validate these assumptions. To further investigate the cause of the relatively lower expression of lncRNAs in LoVo cells, we examined the α-2,6-sialic acid modification, a major factor contributing to cancer hypersialylation (23, 83). The results of flow cytometry and confocal microscopy showed that LoVo cells had a significantly lower α-2,6-sialylation on the cell surface than either HCoEpiC or HCT116 cells. There are also reports about the different glycan modification patterns in LoVo and HCT116 cells (77, 84). Considering the positive expression correlation of most lncRNAs in the signature with sialyltransferases, different sialylation patterns of different cells could explain the relatively poor expression of *ENTPD1-AS1*, *LINC00261*, *LOC100507403*, and *ITGB8-AS1* in Lovo cells.

Some limitations should be addressed in the current study. First, most GEO datasets containing CRC patient data used platform GPL24676 and platform GPL570 and both lack tracks for *ITGB8-AS1* or *AC092687.3*. This can be explained by the different coverage between RNA sequencing and microarrays and shows that the importance of lncRNAs remains masked with the limitation of available technology. Due to the lack of lncRNA expression profiles and complete survival time for CRC, the sialylation-related lncRNA signature was validated using the GSE147602 and GSE198103 datasets. GSE147602 used a new platform, GPL21047, with the metastasis status rather than survival condition to demonstrate the value of our signature. Although we included data for a total of 592 CRC patients from both RNA sequencing and microarray platforms, more independent CRC cohorts and a prospective study are needed for validating the risk signature in the future. Second, although the expression levels of all seven lncRNAs were assayed by RT-qPCR in the CRC cell lines and normal cells, sufficient clinical samples and corresponding clinical information was not available, and subsequent studies with larger sample sizes would be helpful to provide strong evidence for confirming the prognostic value of our signature. Third, the mechanisms by which these sialylation-related lncRNAs cooperate in affecting the immune landscape and in determining drug sensitivity in CRC remain unclear. The biological functions of these lncRNAs and their interactions with sialylation-related genes will be explored in future studies based on *in vitro* and *in vivo* experiments.

In conclusion, this is the most systemic exploration of the clinical significance of sialylation-related lncRNAs in patients with CRC to date. We successfully developed and validated a novel seven sialylation-related lncRNA signature, which exhibited accurate performance in predicting the prognosis, immune status, and chemotherapy sensitivity in CRC patients. The present study may provide innovative perspectives in clinical outcome prediction for CRC patients and contribute to deepening the theoretical foundation for immunotherapy improvement and individualized antitumor treatment.

## Data availability statement

The original contributions presented in the study are included in the article/**Supplementary Material**. Further inquiries can be directed to the corresponding authors.

## Author contributions

Conception and design: MY and MZ. Administrative support: MY. Provision of study materials or patients: WW, YH, FZ, and ZY. Collection and assembly of data: MZ, SL, TL, and WG. Data analysis and interpretation: MZ, RZ, SL, and ZZ. Article writing: MY and MZ. All authors contributed to the article and approved the submitted version.

## Funding

This work was supported by grants from CAMS Innovation Fund for Medical Sciences (CIFMS) (No. 2021-I2M-1-028) and the Natural Science Foundation of China (NSFC) (No. 81773750).

## Acknowledgments

The authors are thankful for the publicly available databases.

## Conflict of interest

The authors declare that the research was conducted in the absence of any commercial or financial relationships that could be construed as a potential conflict of interest.

## Publisher’s note

All claims expressed in this article are solely those of the authors and do not necessarily represent those of their affiliated organizations, or those of the publisher, the editors and the reviewers. Any product that may be evaluated in this article, or claim that may be made by its manufacturer, is not guaranteed or endorsed by the publisher.
