# Supplementary material for: Characterization of sialylation-related long noncoding RNAs to develop a novel signature for predicting prognosis, immune landscape, and chemotherapy response in colorectal cancer
Source: Front Immunol. 2022 Oct 18;13:994874. doi: 10.3389/fimmu.2022.994874 (PMC9623420; doi:10.3389/fimmu.2022.994874)
Supplement: Supplementary file 1 [file DataSheet_1.docx]

**SUPPLEMENTARY MATERIALS**

**Supplementary Table S1: Sialylation-related mRNA downloaded from MsigDB**

| **Entrez Gene ID** | **Gene Symbol** | **Pathway name** |
| --- | --- | --- |
| 6482 | ST3GAL1 | 1,2,3,5,6,7,8,10 |
| 6483 | ST3GAL2 | 1,2,3,5,6,7,8,11 |
| 55808 | ST6GALNAC1 | 1,2,5,6 |
| 51046 | ST8SIA3 | 1,3,4,5,6,8,10 |
| 7903 | ST8SIA4 | 1,3,4,5,6,8 |
| 8128 | ST8SIA2 | 1,3,4,5,6,8 |
| 6487 | ST3GAL3 | 1,3,5,6,7,8 |
| 256435 | ST6GALNAC3 | 1,3,5,6,8 |
| 27090 | ST6GALNAC4 | 1,3,5,6,8 |
| 30815 | ST6GALNAC6 | 1,3,5,6,8 |
| 338596 | ST8SIA6 | 1,3,5,6,8 |
| 81849 | ST6GALNAC5 | 1,3,5,6,8 |
| 8869 | ST3GAL5 | 1,3,5,6,8 |
| 10825 | NEU3 | 1,3,9 |
| 129807 | NEU4 | 1,3,9 |
| 4758 | NEU1 | 1,3,9 |
| 4759 | NEU2 | 1,3,9 |
| 6484 | ST3GAL4 | 1,5,6,7,10 |
| 10402 | ST3GAL6 | 1,5,6,7 |
| 10610 | ST6GALNAC2 | 1,5,6,10 |
| 6480 | ST6GAL1 | 1,5,6,10 |
| 29906 | ST8SIA5 | 1,5,6 |
| 6489 | ST8SIA1 | 1,5,6 |
| 84620 | ST6GAL2 | 1,5,6 |
| 2720 | GLB1 | 1,7 |
| 26503 | SLC17A5 | 1,11,13 |
| 10020 | GNE | 1 |
| 10559 | SLC35A1 | 1 |
| 140838 | NANP | 1 |
| 54187 | NANS | 1 |
| 5476 | CTSA | 1 |
| 55907 | CMAS | 1 |
| 80896 | NPL | 1 |
| 9334 | B4GALT5 | 2,3,8 |
| 11226 | GALNT6 | 2 |
| 11227 | GALNT5 | 2 |
| 114805 | GALNT13 | 2 |
| 117248 | GALNT15 | 2 |
| 168391 | GALNTL5 | 2 |
| 192134 | B3GNT6 | 2 |
| 2589 | GALNT1 | 2 |
| 2590 | GALNT2 | 2 |
| 2591 | GALNT3 | 2 |
| 26290 | GALNT8 | 2 |
| 2650 | GCNT1 | 2 |
| **Entrez Gene ID** | **Gene Symbol** | **Pathway name** |
| 29071 | C1GALT1C1 | 2 |
| 374378 | GALNT18 | 2 |
| 442117 | GALNTL6 | 2 |
| 50614 | GALNT9 | 2 |
| 51301 | GCNT4 | 2 |
| 51809 | GALNT7 | 2 |
| 55568 | GALNT10 | 2 |
| 56913 | C1GALT1 | 2 |
| 57452 | GALNT16 | 2 |
| 63917 | GALNT11 | 2 |
| 64409 | GALNT17 | 2 |
| 79623 | GALNT14 | 2 |
| 79695 | GALNT12 | 2 |
| 8693 | GALNT4 | 2 |
| 9245 | GCNT3 | 2 |
| 140873 | C20orf173 | 3,5,6,8 |
| 2583 | B4GALNT1 | 3,8 |
| 8705 | B3GALT4 | 3,8 |
| 9331 | B4GALT6 | 3,8 |
| 2760 | GM2A | 3,9 |
| 3073 | HEXA | 3,9 |
| 3074 | HEXB | 3,9 |
| 20 | ABCA2 | 3 |
| 3696 | ITGB8 | 3 |
| 54982 | CLN6 | 3 |
| 4099 | MAG | 4,12 |
| 100049587 | SIGLEC14 | 4 |
| 114132 | SIGLEC11 | 4 |
| 2219 | FCN1 | 4 |
| 27036 | SIGLEC7 | 4 |
| 27180 | SIGLEC9 | 4 |
| 27181 | SIGLEC8 | 4 |
| 284369 | SIGLECL1 | 4 |
| 375790 | AGRN | 4 |
| 400709 | SIGLEC16 | 4 |
| 6401 | SELE | 4 |
| 6403 | SELP | 4 |
| 8778 | SIGLEC5 | 4 |
| 89790 | SIGLEC10 | 4 |
| 89858 | SIGLEC12 | 4 |
| 933 | CD22 | 4 |
| 9370 | ADIPOQ | 4 |
| 945 | CD33 | 4 |
| 946 | SIGLEC6 | 4 |
| 10331 | B3GNT3 | 7 |
| 10678 | B3GNT2 | 7 |
| 11041 | B4GAT1 | 7 |
| 11046 | SLC35D2 | 7 |
| 23563 | CHST5 | 7 |
| 2799 | GNS | 7 |
| 4166 | CHST6 | 7 |
| 79369 | B3GNT4 | 7 |
| 8534 | CHST1 | 7 |
| 8702 | B4GALT4 | 7 |
| 93010 | B3GNT7 | 7 |
| 9435 | CHST2 | 7 |
| 10050 | SLC17A4 | 11,13 |
| 10246 | SLC17A2 | 11,13 |
| 10786 | SLC17A3 | 11,13 |
| 6568 | SLC17A1 | 11,13 |
| 25999 | CLIP3 | 12 |
| 54749 | EPDR1 | 12 |
| 5660 | PSAP | 12 |
| 65078 | RTN4R | 12 |

**Pathway name**:

1. REACTOME_SIALIC_ACID_METABOLISM
2. KEGG_O_GLYCAN_BIOSYNTHESIS
3. GOBP_GANGLIOSIDE_METABOLIC_PROCESS
4. GOMF_SIALIC_ACID_BINDING
5. GOBP_SIALYLATION
6. GOMF_SIALYLTRANSFERASE_ACTIVITY
7. GOBP_KERATAN_SULFATE_METABOLIC_PROCESS
8. GOBP_GANGLIOSIDE_BIOSYNTHETIC_PROCESS
9. GOBP_GANGLIOSIDE_CATABOLIC_PROCESS
10. GOBP_PROTEIN_SIALYLATION
11. GOBP_SIALIC_ACID_TRANSPORT
12. GOMF_GANGLIOSIDE_BINDING
13. GOMF_SIALIC_ACID_TRANSMEMBRANE_TRANSPORTER_ACTIVITY

**Supplementary Table S2: Clinicopathological characteristics of TCGA-COAD and TCGA-READ cohorts**

| **Factor** | | **N/percentage(%)** | | |
| --- | --- | --- | --- | --- |
|  |  | **Training** | **Testing** | **Combined** |
|  |  | **N=405** | **N=138** | **N=543** |
| **Age** | ＞65 | 227(56.0%) | 77(55.8%) | 320(58.9%) |
|  | ≤65 | 178(44.0%) | 61(44.2%) | 223(41.1%) |
| **Gender** | Male | 221(54.6%) | 75(54.3%) | 247(54.5%) |
|  | Female | 184(45.4%) | 63(45.7%) | 296(45.5%) |
| **Clinical stage** | Ⅰ | 70(17.3%) | 25(18.1%) | 95(17.5%) |
|  | Ⅱ | 151(37.3%) | 52(37.7%) | 203(37.4%) |
|  | Ⅲ | 123(30.4%) | 41(29.7%) | 164(30.2%) |
|  | Ⅳ | 61(15.1%) | 20(14.5%) | 81(14.9%) |
| **M stage** | M_0_ | 311(76.8%) | 105(76.1%) | 416(76.6%) |
|  | M_1_ | 60(14.8%) | 19(13.8%) | 79(14.5%) |
|  | M_x_ | 34(8.4%) | 14(10.1%) | 48(8.8%) |
| **N stage** | N_0_ | 229(56.6%) | 78(56.5%) | 307(56.5%) |
|  | N_1_ | 98(24.2%) | 36(26.1%) | 134(24.7%) |
|  | N_2_ | 78(19.3%) | 24(17.4%) | 102(18.8%) |
| **T stage** | T_1_ | 10(2.5%) | 6(4.3%) | 16(2.9%) |
|  | T_2_ | 73(18.0%) | 22(15.9%) | 95(17.5%) |
|  | T_3_ | 282(69.6%) | 92(66.7%) | 374(68.9%) |
|  | T_4_ | 40(9.9%) | 18(13.0%) | 58(10.7%) |

COAD: colon adenocarcinoma

READ: rectal adenocarcinoma

**Supplementary Table S3: Primers used for RT-qPCR.**

| **Genes** | **Sequence（5'-3')** |
| --- | --- |
| ZEB1-AS1 | ACTGGTAGCCCAAATCTTCTAAC |
|  | GTCTGTTTTCTTTCCAGTCCAGG |
| LOC100506691 | CACCGGCCCTGAACTCTTT |
|  | GCCTGTTGCAGTTCTTTGCT |
| LOC100507403 | AATAACCCCCGTGCTTCATGT |
|  | CAGGGTTAGGGGCTCATTCA |
| AC092687.3 | TTCTCCAGCGGGGATAATGC |
|  | CTGCGAGGAGGGAATGTGAA |
| LINC00261 | GCAATCCCCTCCTGAGCATT |
|  | CTCCACGGGCTACCAAATGT |
| ITGB8-AS1 | AACGGGGTGCACAGGATTAG |
|  | GCAGATCCAGGGCTCGTTTA |
| ENTPD1-AS1 | GGTGAAGATGCTCAACCTTTGAT |
|  | TCCCCGTGAATGTTCCTCTG |
| GAPDH | CTGACTTCAACAGCGACACC |
|  | TGAGCTTGACAAAGTGGTCGT |

**Supplementary Figure S1**


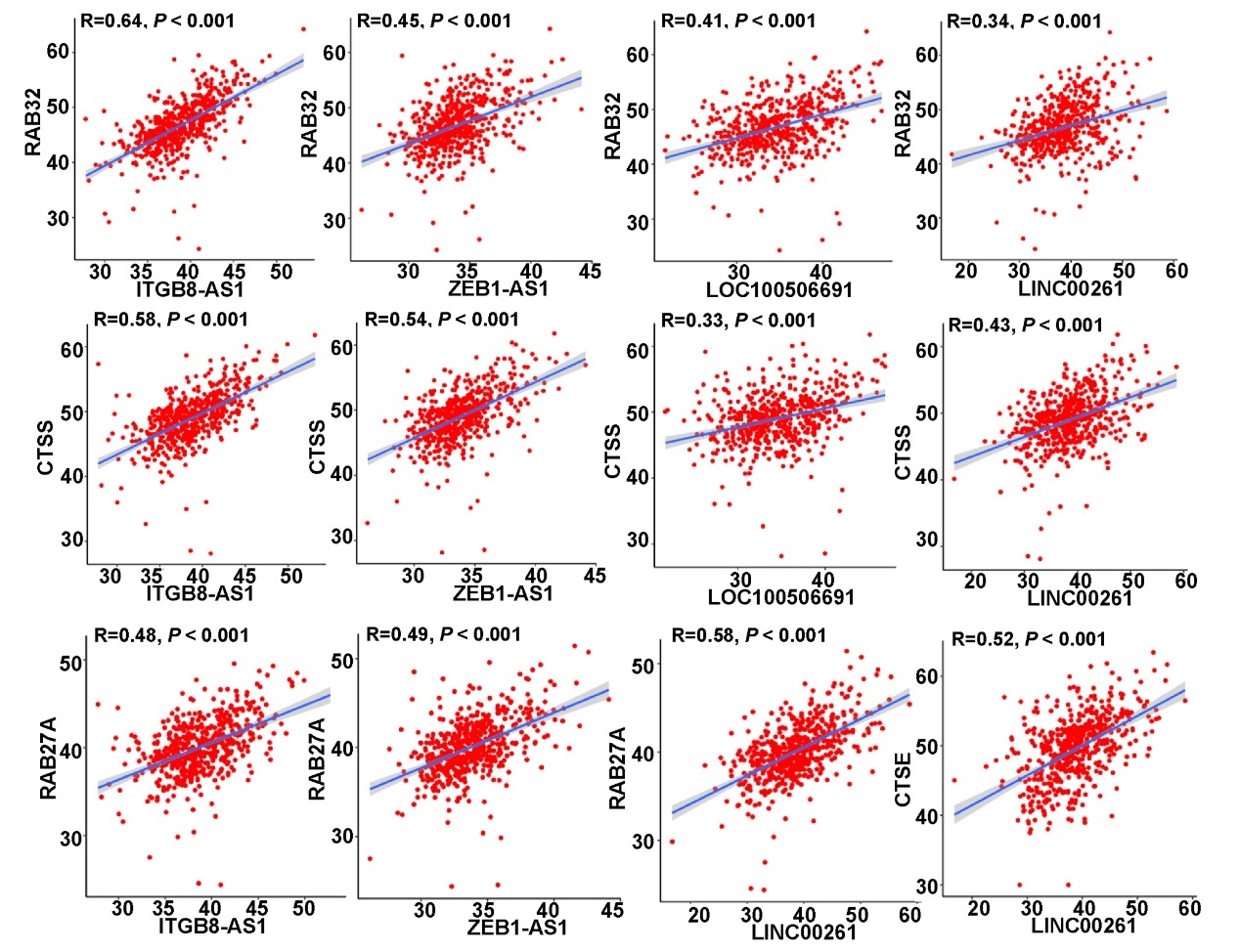


**Supplementary Figure S1** Scattered dot plots of the correlation between the expression of target lncRNAs and the expression of MHC family genes (RAB32, CTSS, RAB27A, CTSE). The Spearman correlation coefficients (R) and corresponding *P* values are shown.

**Supplementary Figure S2**


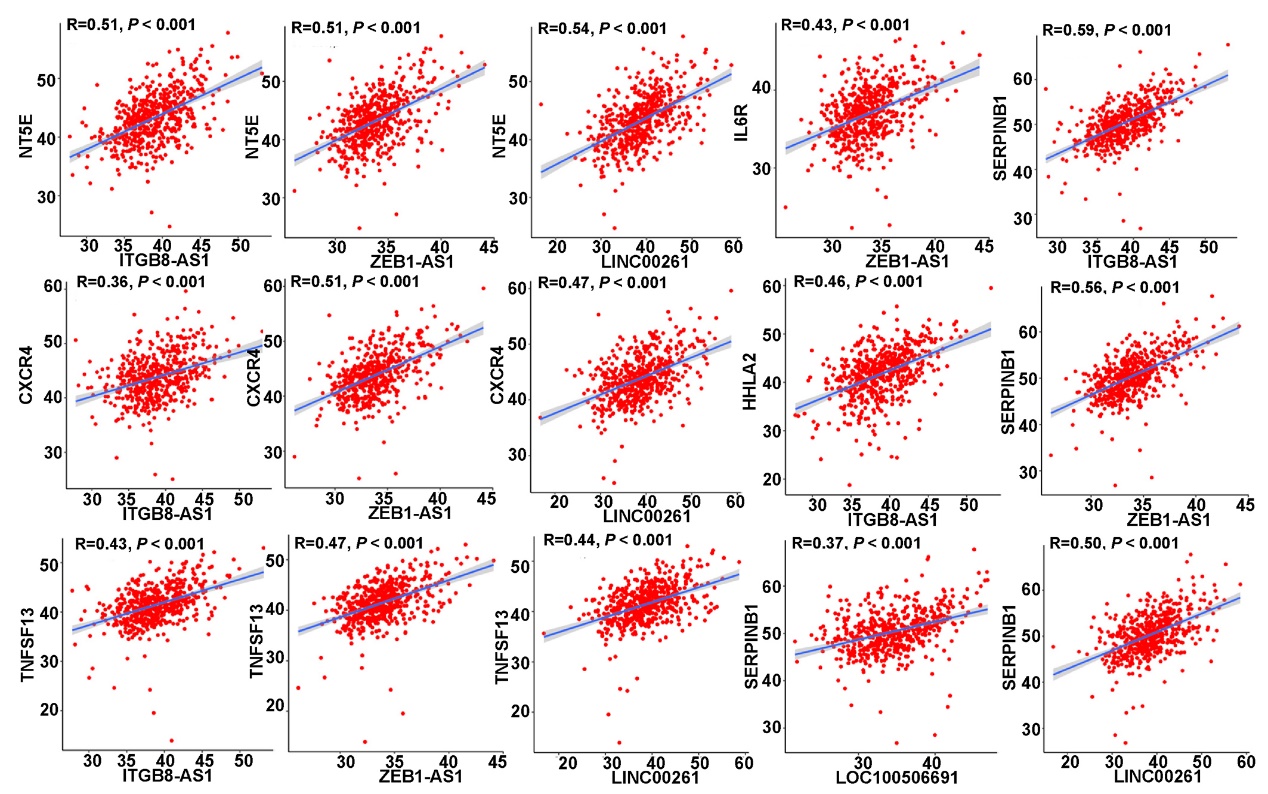


**Supplementary Figure S2** Scattered dot plots of the correlation between the expression of target lncRNAs and the expression of immune stimulators (NT5E, IL6R, CXCR4, HHLA2, TNFSF13) or SERPINB1. The Spearman correlation coefficients (R) and corresponding *P* values are shown.
